# Editorial: Genome-based nutrition strategies for preventing diet-related chronic diseases: where genes, diet, and food culture meet, volume II

**DOI:** 10.3389/fnut.2025.1755919

**Published:** 2026-01-19

**Authors:** Sonia Roman, Claudia Ojeda-Granados, Arturo Panduro

**Affiliations:** 1 Department of Genomic Medicine in Hepatology, Civil Hospital of Guadalajara, Guadalajara, Jalisco, Mexico; 2 Health Sciences Center, University of Guadalajara, Guadalajara, Jalisco, Mexico; 3 Department of Medical and Surgical Sciences and Advanced Technologies “GF Ingrassia”, University of Catania, Catania, Italy

**Keywords:** chronic diseases, dietary patterns, food culture, gene polymorphisms, gene-environmental mismatch, lifestyle factors, personalized medicine, personalized nutrition

## Introduction

Volume I of “*Genome-based Nutrition Strategies for Preventing Diet-related Chronic Diseases: Where Genes, Diet, and Food Culture Meet”* laid the groundwork for genome-based nutrition, highlighting how genetic variability, dietary choices, and cultural practices interact to influence chronic disease risk (1). These elements together form the foundation for personalized, culturally sensitive, and scientifically sound nutrition strategies. Volume II advances this concept by presenting empirical research and translational approaches that demonstrate the practical power of the gene–diet–food culture triad. This triad comprises genetic profiles as the biological basis for nutrigenomics, evidence of specific genetic polymorphisms that predispose populations to metabolic disorders, and the roles of regional foods and food cultures in buffering or amplifying genetic risks.

A key aspect of this framework is distinguishing between “regional foods”—traditional, locally adapted diets that align with ancestral genetic profiles and often provide protection against chronic diseases—and “mismatch foods,” which are energy-dense, processed, and disruptive to ancestral gene–diet alignments, increasing vulnerability to metabolic disorders. Food culture, including traditions and social contexts, further mediates exposure to either protective or mismatch foods, influencing whether traditional diets are preserved or replaced.

This Research Topic brings together experts from genetics, nutrition, epidemiology, and clinical medicine, integrating molecular genetics, public health, clinical nutrition, and bioinformatics to reflect the complexity of nutritional genomics. The contributing studies cover diverse geographic regions—including Asia, North America, Europe, and the Middle East—providing a global perspective on gene–diet interactions. Notably, the research converges on themes of precision medicine, personalized nutrition, metabolic disease prevention, and dietary adaptation based on genetic profiles.

This Research Topic highlights how the gene–diet–food culture triad operates across different populations, emphasizing that precision nutrition should realign genes and regional foods while minimizing the mismatch introduced by globalization.

## Saudi Arabia: *TCF7L2* variant and the impact of nutrition transition

In Saudi Arabia, genetic vulnerability combined with rapid dietary and cultural changes has led to a metabolic health crisis, with metabolic syndrome affecting over 30% of adults. Al-odinan et al. examined the *TCF7L2* rs7903146 (C/T) variant, which is strongly associated with type 2 diabetes (T2D). Carriers of the TT genotype showed higher energy intake, increased waist circumference, and altered insulin responses, indicating greater susceptibility to metabolic disorders.

Historically, Saudi diets centered on dates, legumes, whole grains, and lean meats, which were well matched to the population's metabolic needs and helped buffer genetic risks. However, rapid urbanization has introduced Westernized foods—processed items, sugary drinks, and high saturated fat—that disrupt these protective traditions and amplify genetic vulnerabilities.

## Mexican Americans: *FADS* variants and omega-3 deficiency

Blomquist et al. studied *FADS* and *ELOVL* gene variants in Mexican Americans with significant Indigenous ancestry. The ancestral haplotype impairs omega-3 high unsaturated fatty acid (HUFA) biosynthesis, increasing the risk for insulin resistance and high triglycerides. Traditional Indigenous diets rich in fish and marine fats supported genetic adaptations and maintained a healthy HUFA balance. Westernized diets, high in omega-6 vegetable oils but low in omega-3, disrupt this balance and worsen cardiometabolic risk.

Cultural pressures and acculturation replace protective marine foods with processed oils, increasing exposure to mismatch foods. These gene–diet interactions suggest the need for targeted omega-3 supplementation strategies, especially for communities facing limited access to protective foods and a disproportionate burden of metabolic diseases.

## Mexico: *FTO* variants in Amerindian populations

Sepúlveda-Villegas et al. explored the *FTO* rs9939609 polymorphism in West Mexican populations. The T allele is prevalent among Amerindians, while the A allele is more common in mestizos with higher European ancestry. The TT genotype correlates with insulin resistance, elevated triglycerides, and hyperglycemia.

Traditional Amerindian diets—corn, beans, squash, wild greens—were low in energy density and protective. Modern diets, characterized by ultra-processed and high-carbohydrate foods, overwhelm ancestral adaptations and increase disease risk, especially in urbanized populations. This nutrition transition highlights the necessity for policies that address Mexico's genetic diversity and unequal exposure to nutritional risks, ensuring that protective diets are prioritized for vulnerable groups.

## Korea: nutrient-specific gene–diet interactions

Park et al. conducted a genome-wide study in Koreans, identifying *MELTF* rs73893755 and *TRIM25* rs139560285 variants that elevate T2D risk when interacting with nutrient intake. Traditional Korean diets, rich in vegetables and fermented foods with moderate cholesterol, help buffer genetic risk. Consumption of modern diets with excess cholesterol and vitamin A increases susceptibility.

Korean Daily Recommended Intakes (DRIs) represent cultural dietary baselines, but modern dietary transitions challenge adherence to these recommendations, increasing exposure to mismatch foods and genetic risk.

## South Asia vs. Arctic: comparative adaptations

Pathak et al. compared genetic adaptations in populations from tropical South Asia and the Arctic. South Asians are genetically adapted to starch-rich, vegetarian diets (e.g., high *AMY1A* copy number), while Arctic populations have adapted to high-fat diets from marine resources and reindeer (e.g., *FADS, CPT1A, TBC1D4*). Traditional dietary practices—religious vegetarianism in South Asia and subsistence hunting/herding in the Arctic—preserved protective diets.

The introduction of Westernized foods—refined carbohydrates in South Asia and processed fats in the Arctic—disrupts ancestral gene–diet alignments, undermining protective adaptations and increasing metabolic disease risk.

## China: gestational diabetes and rs6127416

Li et al. identified rs6127416 as a genetic determinant of gestational diabetes in southern Chinese women. The AA genotype is associated with increased risk, especially alongside elevated pre-pregnancy BMI and fasting glucose. Traditional maternal diets emphasizing rice, vegetables, and balanced energy intake can buffer against genetic risk.

However, urban dietary shifts toward high-calorie, processed foods exacerbate genetic susceptibility. Antenatal care and dietary traditions mediate exposure, but adherence to protective diets is challenged by modern lifestyles.

## China: multigene synergism and obesity

Yang et al. reviewed multiple SNPs in genes *(FTO, MC4R, BDNF, ADIPOQ, TCF7L2, TRHR, RASAL1*) that contribute to obesity in Chinese adults, highlighting multigene synergistic effects and sex-specific differences. The interaction between genetic risk and regional dietary patterns, shaped by recent cultural transitions, drives heterogeneity in obesity prevalence. Traditional southern Chinese diets—plant-based, high in vegetables and grains—buffer against genetic risk, while Westernized diets high in fat, sugar, and processed foods increase obesity rates. Regional and cultural differences mediate dietary exposure, determining whether populations benefit from protective foods or are exposed to mismatch diets.

## Final considerations

The studies that comprise Volume II of our Research Topic make it clear that metabolic diseases result from a complex interplay between our ancestral genetic makeup and modern dietary environments, not simply excess calories or inactivity. Genetic effects are shaped by regional diets and cultural practices. Thus, framing each study within the gene–diet–food culture triad highlights the need for nutrition strategies that respect cultural and genetic backgrounds. This framework distinguishes protective foods from mismatch foods; mismatch foods disrupt ancestral health alignments and increase vulnerability to conditions like insulin resistance, dyslipidemia, and obesity, while traditional foods act as buffers by providing nutrients suited to ancestral adaptations. Food culture influences dietary exposure and sustainability, determining which foods dominate a population's diet.

Nutritional genomics, through the lens of this triad, helps explain obesity, diabetes, and related conditions, emphasizing the need for interdisciplinary and culturally sensitive policies and regional clinical guidelines. As this science progresses, it supports genome-based nutrition strategies that reconnect genetic profiles with protective regional foods and traditions, minimizing mismatch diet exposure. By honoring genetic and cultural backgrounds, we can develop nutrition approaches that better address metabolic disorders and preserve ancestral health alignments, making precision nutrition a path to restoring balance and resilience in diverse communities (2).
